# The Effect of Metaphylactic Use of Tildipirosin for the Control of Respiratory Disease in Long-Distance Transported Dairy Calves

**DOI:** 10.3389/fvets.2020.00632

**Published:** 2020-09-08

**Authors:** Maria Luiza Celestino, Leticia Fernandes, Paulo Roberto Menta, Daniela Paiva, Thiago Lauro Ribeiro, Thiago Silva, Todd R. Bilby, Rafael C. Neves, Michael A. Ballou, Vinicius S. Machado

**Affiliations:** ^1^Department of Veterinary Sciences, College of Agricultural Sciences and Natural Resources, Texas Tech University, Lubbock, TX, United States; ^2^Department of Animal Science, School of Animal Science and Food Engineering, University of São Paulo, São Paulo, Brazil; ^3^Merck Animal Health, Madison, NJ, United States; ^4^Department of Veterinary Clinical Sciences, College of Veterinary Medicine, Purdue University, West Lafayette, IN, United States

**Keywords:** dairy calves, metaphylaxis, BRD, tildipirosin, transportation

## Abstract

The objective of this study was to evaluate the efficacy of two metaphylactic strategies using tildipirosin for the control of bovine respiratory disease (**BRD**) in dairy calves transported to a heifer raising facility within their first week of life. A total of 2,100 calves were enrolled in the study. Animals were transported for ~1,715 km, from dairies located in Minnesota to a calf raising facility located in New Mexico, where they were housed in individual hutches until weaning. Three days after arrival, calves were randomly allocated into three groups: (1) META1: single subcutaneous (SQ) injection of tildipirosin (Zuprevo™, Merck Animal Health) at enrollment at 4 mg/kg; (2) META2: SQ injection of tildipirosin at enrollment and 17 days later; (3) CON: untreated controls. The BRD incidence was 11.4, 10.8, and 9.4% for calves enrolled in the CON, META1, and META2, respectively (*P* = 0.44). Lung lesions diagnosed through ultrasonography was found in 21.0, 21.0, and 21.8% of calves enrolled in CON, META1, and META2, respectively (*P* = 0.99). Mortality tended to be greater for CON calves in comparison to META2 calves (1.5 vs. 0.6%, *P* = 0.06), but did not differ between calves enrolled in CON and META1 groups (1.5 vs. 1.2%, *P* = 0.55). Growth was not affected by metaphylaxis. The average daily gain for calves enrolled in CON, META1, and META2 was 517, 518 and 525 g, respectively (*P* = 0.25). Blood analysis revealed that some of the markers of inflammation assessed were influenced by metaphylaxis. At 27 days after enrollment, META2 calves had decreased concentrations of haptoglobin, serum amyloid A, and aspartate aminotransferase, compared to CON calves (*P* < 0.05). Additionally, CON calves had increased concentrations of globulins and lower albumin to globulin ratio than META2 calves at the end of the weaning period (*P* < 0.05). In conclusion, tildipirosin metaphylaxis did not decrease the incidence of BRD nor did it have an impact on weight gain. However, metaphylaxis with two injections of tildipirosin at enrollment and 17 days later tended to reduce mortality and improved the systemic inflammatory status of calves during the preweaning period.

## Introduction

Bovine respiratory disease (**BRD**) is a highly prevalent and multifactorial illness responsible for production losses in pre-weaned dairy calves. Clinical signs associated with BRD are nasal and ocular discharge, cough, fever, and droopy ears (1, 2). According to the United States Department of Agriculture (**USDA**) National Animal Health Monitoring Survey conducted in 2014, BRD affected 27% of pre-weaned calves and caused 14.1% of deaths (3). Recently, a study performed in California dairies between 2015 and 2016, reported 22.8% BRD incidence in pre-weaned dairy calves totaling 19.3% case fatality rate (4). The short-term economic impact of BRD on farm operations are due to labor (i.e., for disease detection and care of sick animals), medications, veterinary fees, and replacement of dead animals (5, 6). Dubrovsky et al. (4), calculated short-term cost of treating recurrent cases of BRD in dairy calves was $42.15 per calf. Additionally, the long-term costs of BRD are complex and involve impaired performance of animals even when they have received treatment (7, 8). These animals undergo delayed growth during the pre-weaning period (7, 9), decreased reproductive performance (10), increased chance of leaving the herd prior to first calving (7, 8, 11), increased age at first calving (11) and decreased milk production during first lactation (8).

The etiopathogenesis of BRD involves an interaction between host and environmental factors, stressors, pathogens and management practices (12, 13). The disease is usually initiated by a stressful event (i.e., transportation, comingling) followed by a viral or bacterial infection, which predisposes the animals to bacterial infections (14, 15). Viruses such as bovine viral diarrhea virus (BVDV), bovine respiratory syncytial virus (BRSV), parainfluenza type 3 virus (PI-3), bovine corona virus (BCV), bovine adeno- virus (BAV) and bovine herpes virus 1 (BoHV-1) have been described as causative agents of the BRD complex (15, 16). The most common bacterial agents associated with BRD cases are *Mannheimia haemolytica, Pasteurella multocida, Histophilus somni*, and *Mycoplasma bovis*, with the most predominant pathogen being *Mannheimia haemolytica* (7, 15). These pathogens can be found in the upper respiratory tract of both healthy and diseased calves (17). Thus, the onset of the disease will be dependent on bacterial load and risk factors, such as season of birth, failure of transfer of passive immunity, and occurrence of other diseases within the first 14 days of life (18, 19). Also, commingling of animals and long-distance transportation can increase the risk for BRD (7, 20, 21). In the modern U.S. dairy industry, 10% calves are raised in specialized facilities, where calves are acquired from different sources and are transported for long periods of time, which are known stressors leading to increased BRD risk (22).

To minimize deleterious impacts of BRD, metaphylactic antimicrobial administration before the main peak of BRD incidence is a common management practice to reduce pathogen load in a high-risk population (12). The anti-infective tildipirosin (Zuprevo™, Merck Animal Health) is a long-acting macrolide that is indicated for the treatment and control of BRD in high risk cattle. The pharmacokinetics properties of tildipirosin include rapid distribution to lung tissue and bronchial fluid with a long half-life, which leads to a sustained concentration of the macrolide in the lower respiratory tract (23). Reports regarding the effectiveness of the metaphylactic use of tildipirosin to control BRD in high-risk calves have been inconsistent. Metaphylactic use of tildipirosin at arrival did not reduce the number of BRD treated cases in veal calves (13). However, others have shown that metaphylactic injections of tildipirosin reduces the incidence of pneumonia and otitis during the pre-weaning period of dairy calves housed in group pens (9). The efficacy of tildipirosin to control and mitigate the deleterious effects of BRD in dairy calves transported to calf raising facilities are unknown. The objective of this study was to evaluate the effect of two metaphylactic strategies using tildipirosin in the incidence of BRD, growth, and mortality of dairy calves originating from multiple sources and following long transport time within the first week of life (i.e., high risk).

## Materials and Methods

All activities performed in this study were reviewed and approved by the Texas Tech University Institutional Animal Care and Use Committee (#18081-10).

### Animals and Facilities

The study was conducted in a commercial heifer raising facility located in eastern NM, from January 11, 2019 to July 15, 2019. Calves were born in 13 different farms located in Minnesota. General management practices of these farms included immediate separation of calves from their dams and feeding of 4 L of pasteurized (60°C for 60 min) pooled colostrum within the first 6 h of life. Total serum protein was assessed for a subset of calves by farm employees to evaluate and ensure proper colostrum management. Within the first week of life (mean ± SD = 3.78 ± 1.3 days of life), calves were transported from their farm of origin to the calf raising facility located in NM. The approximate transportation distance was 1,715 km. At arrival, calves were individually housed in hutches. Whole milk was fed twice a day (4 L/d), water and calf starter were offered ad libitum during the pre-weaning period. Calves were vaccinated at birth intranasally with BRSV, IBR, and PI3 (Inforce 3, Zoetis, MI), and at 30 days of age and at weaning, with a *Mannheimia haemolytica* bacterin-toxoid bacteria (One Shot, Zoetis, MI).

### Treatment Allocation, Data Collection, and Case Definition

Calves were enrolled in the study at 3 days after arrival and were randomly allocated into three different groups: (1) CON: untreated controls, *n* = 700; (2) META1: single SQ injection of tildipirosin at enrollment, *n* = 700; and (3) META2: one SQ injection of tildipirosin at enrollment and a subsequent SQ tildipirosin injection 17 days later, *n* = 700. Tildipirosin treatments followed the label dose of 4 mg/kg of body weight. Calves were included in the study if they did not present clinical signs associated with BRD, such as ocular or nasal discharge, ear droop, cough, or rectal temperature ≥ 39.2°C. All animals were tested for BVD at enrollment. Fresh skin samples (ear notch) were submitted to the Texas A&M Veterinary Medical Diagnostic Laboratory in Amarillo, TX and tested using the antigen capture ELISA method. Persistently infected animals were excluded from the study. Animals that had been previously treated with antibiotics for BRD or other conditions were not eligible to be enrolled in the study.

Calves were visually inspected by the research team members three times per week (on a M-W-F basis) from enrollment until weaning (60 days of life). The research team used a systematic scoring system developed for the assessment of BRD in pre-weaned dairy calves (2). This validated scoring system assesses six clinical signs (cough, eye discharge, abnormal respiration, nasal discharge, ear droop or head tilt, and rectal temperature ≥ 39.2°C: Cough = 2 points, Eye discharge = 2 points, Fever (≥ 39.2°C) = 2 points, Abnormal respiration = 2 points, Nasal discharge = 4 points, Ear droop or head tilt = 5 points. A total score of five points or higher characterizes a BRD case and treatment was warranted. To achieve blinding of research and farm personnel, treatment allocation and administration were performed by a veterinarian from the research team in the mornings, and BRD scoring for diagnosis was performed by another veterinarian from the research team (unaware of treatment assignment) in the afternoons. In addition to the research group monitoring and scoring recording, animals were visually monitored daily by trained farm employees following the same BRD scoring system utilized by the research team. Farm personnel was also blinded to treatments. Animals diagnosed with BRD were treated with 40 mg/kg florfenicol and 2.2 mg/kg flunixin meglumine (Resflor Gold^®^, Merck Animal Health, NJ). Treated animals had a 4-day post-treatment interval, when they were not eligible to receive subsequent treatment, unless authorized by the herd veterinarian. If clinical signs persisted after 4 days of initial treatment, animals were re-treated with a different drug class (e.g., Enrofloxacin, Baytril^®^ 100, Bayer, NJ).

At enrollment and weaning (60 days of life), ultrasonography of the lungs was assessed for a random subset of 200 calves per treatment. Thoracic ultrasonography was performed by a trained veterinarian using an Ibex-pro device with a 6.2-MHz linear transducer (E.I. Medical Imaging, Loveland, CO). The examination of the lungs was carried out by a dorsal to ventral screening of the thorax. The area from the 1st to the 10th intercostal spaces was screened on the right side of the thorax, and from the 3rd to the 9th on the left side. Consolidation of the lungs was detected based on heterogeneous hypoechoic area in the absence of the pleural surface clear line. Body weight measurements were assessed at enrollment and at the end of the study period (49 days after enrollment) using a digital scale (Calf Cart™, Raytec^®^, Ephrata, PA). These measurements were used to calculate the average daily gain (ADG) during the study period (final weight–initial weight/days in study).

Data regarding mortality, source (farm of origin), total serum protein, date of birth, dam's parity, dam's gestation length were extracted from the farms' database software (DairyComp 305, Valley Agricultural Software, Tulare, CA).

### Blood Sampling and Analysis

Blood was collected for a random subset of 100 calves per treatment group to determine evidence of stress and inflammation. Blood samples were collected at enrollment, 10, 27, and 49 days later by jugular venipuncture using a Vacutainer tube without anticoagulant and a Vacutainer tube with EDTA, and a 20-gauge ×2.54-cm Vacutainer needle (Becton, Dickinson and Company, Franklin Lakes, NJ). After collection, tubes were immediately placed in a cooler containing iced water and transported to Texas Tech University (Lubbock, TX) within 2 h after collection for processing. Blood samples collected without anticoagulant were centrifuged for serum separation, and frozen at −80°C. Samples collected with EDTA were evaluated for complete blood cell (CBC) counts, using a hematology analyzer (IDEXX Procyte DX, Westbrook, ME).

Serum haptoglobin (Hp) concentration was determined using a colorimetric assay via quantification of the haptoglobin/hemoglobin complex by the estimation of differences in peroxidase activity (24). Assays were performed in 16 × 100 borosilicate tubes. Briefly, 5 μL of serum sample or deionized water (blank) were added to 7.5 mL of a solution containing 0.6 g/L of O dianisidine, 13.8 g/L of sodium phosphate monobasic, and 0.5 g/L EDTA (pH = 4.1). Immediately, 25 μL of 0.3 g/L bovine hemoglobin solution was added to each assay, followed by a water bath incubation at 37°C for 45 min. After incubation, 100 μL of freshly prepared 156 mM hydrogen peroxidase solution was added to each assay. Samples were incubated at room temperature for 60 min. Then, 200 μL of each assay was transferred to a 96-well polystyrene flat-bottom microplate. Optical density at 450 nm was measured on the Epoch2 Microplate Spectrophotometer (BioTek, Winoosk, VT). Finally, the final OD of each assay was subtracted by the blank assay OD. Optical density data was converted to a concentration unit (μg/mL) using standard curves generated by serial dilutions of a sample of known concentration determined by a commercially available ELISA kit following the manufacturer's instructions (Life Diagnostics, West Chester, PA) as previously described (25). The intra and inter-assay CV for serum Hp were 6.9 and 7.7%, respectively. Serum amyloid A (SAA) was determined by a commercially available ELISA kit following the manufacturer's instructions (Life Diagnostics, West Chester, PA). The intra and inter-assay CV for serum Hp were 5.2 and 7.3%, respectively. Samples were analyzed for Zinc concentration using a chemistry analyzer (RX Daytona; RANDOX Laboratories, Crumlin, UK) in a single assay, and the intra-assay CV was 1.9%.

A 0.5 mL aliquot of serum was submitted to the Texas A&M Veterinary Medical Diagnostic Laboratory for ruminant chemistry profile (total protein, albumin, albumin to globulin ratio, globulin, glucose, blood urea nitrogen, calcium, phosphorus, creatinine kinase, total bilirubin, aspartate aminotransferase, gamma-glutamyl transferase, magnesium, sodium, potassium, chloride, and glutamate dehydrogenase activity).

### Sample Size Calculation

Based on previous year BRD incidence data from the studied herd, baseline incidence of BRD in pre-weaned calves housed in hutches was anticipated to average 15%, with an assumption that tildipirosin metaphylaxis would reduce BRD incidence by at least 5%. To detect this reduction, 686 calves per treatment group were needed for a study with 80% power and significant differences declared at α = 0.05. To account for eventual data loss (i.e., calves excluded due to BVD diagnosis) a total of 700 calves per treatment group (three treatment groups, *n* = 2,100 total) was enrolled in the study.

### Statistical Analysis

Descriptive statistics were undertaken using the chi-square and ANOVA functions of JMP 14 (SAS Institute Inc., Cary, NC). To evaluate the effect of metaphylaxis on BRD incidence and presence of lung lesions diagnosed by ultrasonography, two multivariable logistic regressions models were fitted to the data using the GLIMMIX procedure of SAS 9.4 (SAS Institute Inc.). Mortality was evaluated using a multivariable Cox's proportional hazard model (PHREG procedure in SAS). Calves were right-censored if they were alive at the end of the data collection period. The effect of metaphylaxis on growth during the pre-weaning period (ADG) was evaluated using the MIXED procedure of SAS. To evaluate the effect of metaphylaxis on the circulating concentration of metabolic and inflammatory markers, multiple mixed general linear models were fitted to the data using the MIXED procedure of SAS. The data comprised a series of repeated measures of each dependent variable throughout the four blood collection days. To account appropriately for within-calf correlation, the error term was modeled by imposing a first-order autoregressive covariance structure for all models. Visual assessment of the distribution plots of the studentized residuals were used to confirm that the residuals were normally distributed.

For all multivariate models described above, independent variables and their respective interactions were kept when *P* < 0.10. Treatment was forced into all statistical models even in the absence of statistical significance. Age in days at enrollment, body weight at enrollment, dam's parity (primiparous or multiparous), season (Winter or Spring), and rectal temperature at enrollment were offered to all models. Origin of source was included under the STRATA statement in the Cox proportional hazard analyses and as a random variable in all other statistical models described above.

## Results

### Descriptive Statistics

Descriptive statistics on averages for age at enrollment (in days), body weight at enrollment, rectal temperature at enrollment, total serum protein (subset of animals), dam's gestation length, parity of dam, total number of animals enrolled by season, and total number of excluded animals are presented in Table 1. Six animals were excluded from the study because they were diagnosed as BVD-PI. One animal was excluded from the study because a few weeks after enrollment, it was noticed that it was a male calf.

**Table 1:** Descriptive statistics of treatment groups.

	**CON^1^**	**META1^2^**	**META2^3^**	***P***
Average age (days) at enrollment (± SE)	7.8 (0.05)	7.8 (0.05)	7.8 (0.05)	0.96
Average body weight (kg) at enrollment (± SE)	32.7 (0.16)	33.0 (0.16)	32.8 (0.16)	0.46
Average rectal temperature (°C) at enrollment (± SE)	38.7 (0.01)	38.7 (0.01)	38.7 (0.01)	0.98
Average total serum protein g/dL (± SE)^4^	6.5 (0.03)	6.5 (0.03)	6.5 (0.03)	0.25
Average days of gestation of dam (± SE)	278.3 (0.61)	277.0 (0.61)	277.7 (0.61)	0.30
Average parity of dam (± SE)	2.3 (0.02)	2.3 (0.02)	2.3 (0.02)	0.87
Total enrolled animals during winter (%)	330 (47.1)	330 (47.1)	330 (47.1)	1.00
Total enrolled animals during spring (%)	370 (52.9)	370 (52.9)	370 (52.9)	
Total enrolled animals (%)	700 (33.3)	700 (33.3)	700 (33.3)	
Total excluded animals (%)	3 (0.43)	1 (0.14)	3 (0.43)	0.51

1 *CON: untreated controls*.

2 *META1: single SQ injection of tildipirosin (4 mg/kg) at enrollment*.

3 *META2: one SQ injection of tildipirosin at enrollment and a subsequent SQ tildipirosin injection 17 days after the first injection*.

4 *Total serum protein was assessed for 310, 327, and 325 calves enrolled in CON, META1, and META2 treatment groups, respectively*.

### Effect of Metaphylaxis on BRD Incidence and Lung Consolidation Diagnosed Through Thoracic Ultrasonography

Tildipirosin metaphylaxis did not decrease the incidence of BRD during the pre-weaning period of dairy calves (Table 2, *P* = 0.44). The BRD incidence was 11.4, 10.8, and 9.4% for calves enrolled in CON, META1, and META 2, respectively. Similarly, tildipirosin metaphylaxis did not decrease the proportion of calves diagnosed with lung lesions through ultrasonography at weaning (Table 2, *P* = 0.99). The proportion of calves diagnosed with lung consolidation at the end of the study period was 21.0, 21.0, and 21.8% for CON, META1 and META2 calves, respectively.

**Table 2:** Effect of tildipirosin metaphylaxis on the incidence of bovine respiratory disease (BRD), ultrasonographic lung consolidation (ULC) at weaning, mortality, and average daily gain (ADG).

	**CON^1^**	**META1^2^**	**META2^3^**
**BRD**			
Incidence (%)	11.4	10.8	9.4
Odds ratio (95% CI)	*baseline*	0.95 (0.68–1.31)	0.80 (0.57–1.13)
*P*		0.75	0.21
**ULC**			
Affected calves (%)	21.0	21.0	21.8
Odds ratio (95% CI)	*baseline*	1.00 (0.62–1.60)	1.05 (0.66–1.68)
*P*		1.00	0.97
**Mortality**			
Dead calves (%)	1.5	1.2	0.6
Hazard ratio (95% CI)	*Baseline*	0.77 (0.32–1.82)	0.34 (0.11–1.06)
*P*		0.55	0.06
***ADG (g)***	517	518	525
95% CI	(508–525)	(509–526)	(516–533)
*P*		0.84	0.12

1 *CON: untreated controls*.

2 *META1: single SQ injection of tildipirosin (4 mg/kg) at enrollment*.

3 *META2: one SQ injection of tildipirosin at enrollment and a subsequent SQ tildipirosin injection 17 days after the first injection*.

### Effect of Metaphylaxis on Mortality and Average Daily Gain

Although we did not observe treatment differences in lung health outcomes, tildipirosin metaphylaxis at enrollment and 17 days later tended to decrease mortality (Table 2). Hazard of death was 2.94 times higher for CON calves in comparison to META2 calves (*P* = 0.06). However, the hazard of death did not differ between CON and META1 calves (*P* = 0.55). Additionally, tildipirosin metaphylaxis did not influence growth of pre-weaned calves (Table 2, *P* = 0.25). The ADG during the study period for CON, META1, and META2 calves were 517, 518, and 525 g, respectively.

### Effect of Metaphylaxis on Blood Variables

**Figure 1:**
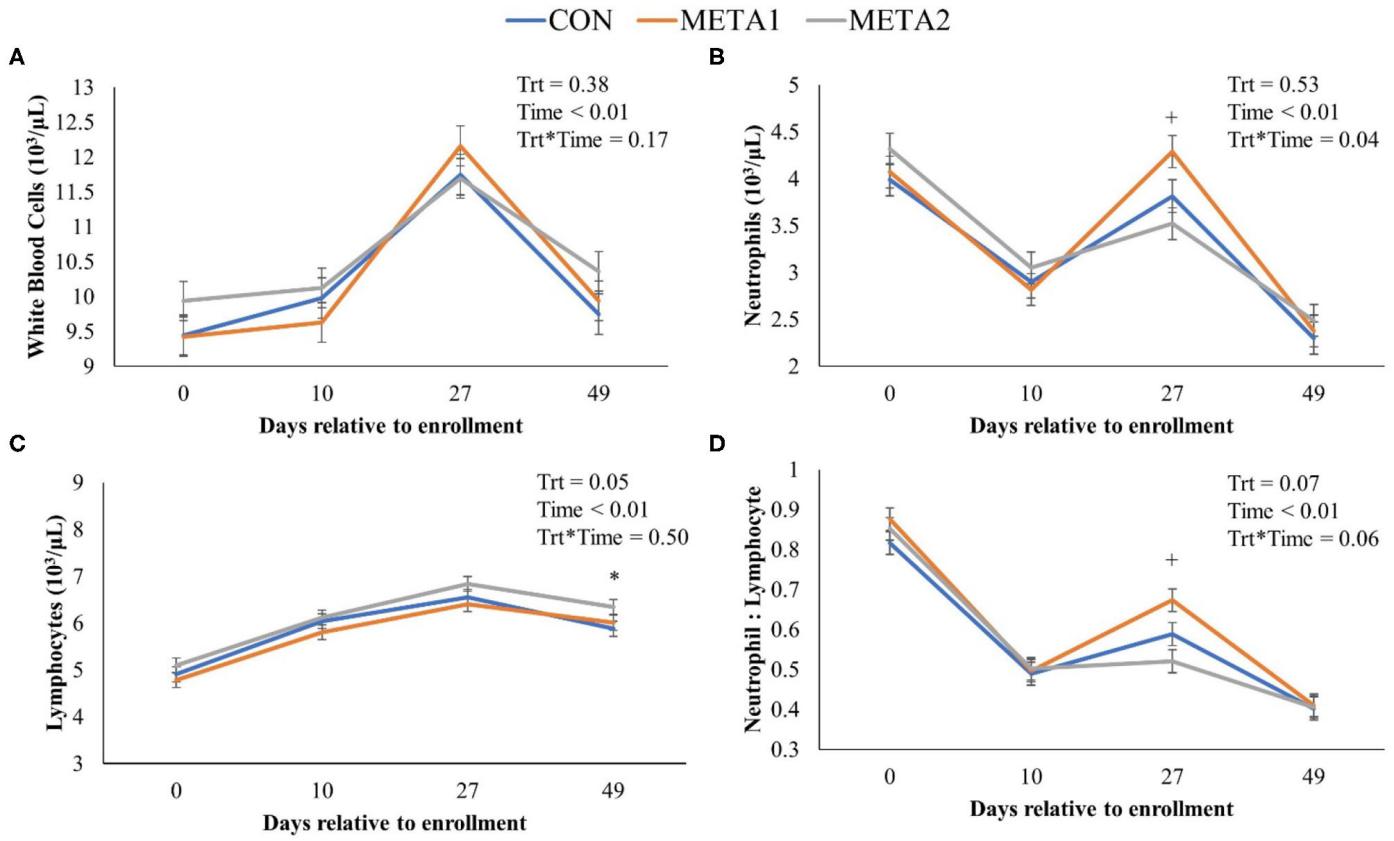
Effect of metaphylaxis on white blood cells **(A)**, neutrophils **(B)**, lymphocytes **(C)**, and neutrophils to lymphocytes ratio **(D)**. A cross (+) or asterisk (^*^) indicates a *P* < 0.05 when comparing CON with META1 or META2, respectively. Calves enrolled in META1 received single SQ injection of tildipirosin (4 mg/kg) at enrollment, calves enrolled in META2 received one SQ injection of tildipirosin at enrollment and a subsequent SQ tildipirosin injection 17 days after the first injection, and CON calves remained untreated.

**Figure 2:**
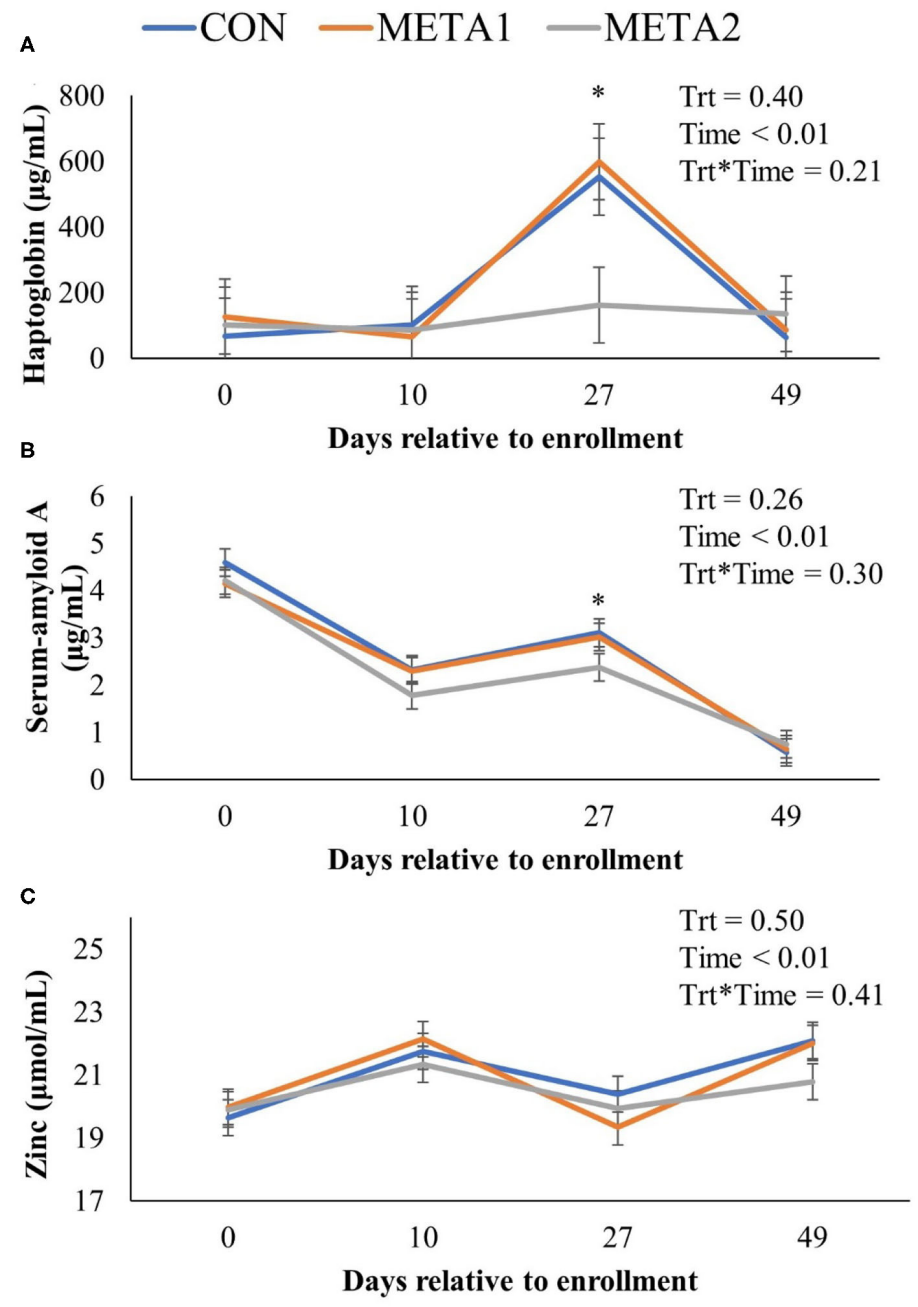
Effect of metaphylaxis on circulating concentrations of haptoglobin **(A)**, serum-amyloid A **(B)**, and zinc **(C)**. An asterisk (^*^) indicates a *P* < 0.05 when comparing CON with META2. Calves enrolled in META1 received single SQ injection of tildipirosin (4 mg/kg) at enrollment, calves enrolled in META2 received one SQ injection of tildipirosin at enrollment and a subsequent SQ tildipirosin injection 17 days after the first injection, and CON calves remained untreated.

The effect of tildipirosin metaphylaxis on white blood cell, neutrophil, and lymphocyte counts, and neutrophil to lymphocyte ratio is depicted in Figure 1. Metaphylaxis did not influence the white blood cell count of calves during the study (*P* = 0.38). However, neutrophils, and neutrophil to lymphocyte ratio at 27 days after enrollment were greater for META1 calves in comparison to CON calves. Additionally, META 2 calves had greater lymphocyte counts than CON calves at the last day of the study. The effect of metaphylaxis on circulating concentrations of Hp, SAA, and zinc is presented in Figure 2. Calves enrolled in META2 had decreased concentrations of the acute phase proteins Hp and serum-amyloid A than CON calves at 27 days after enrollment. Metaphylaxis did not influence the circulating concentrations of zinc throughout the study period.

**Figure 3:**
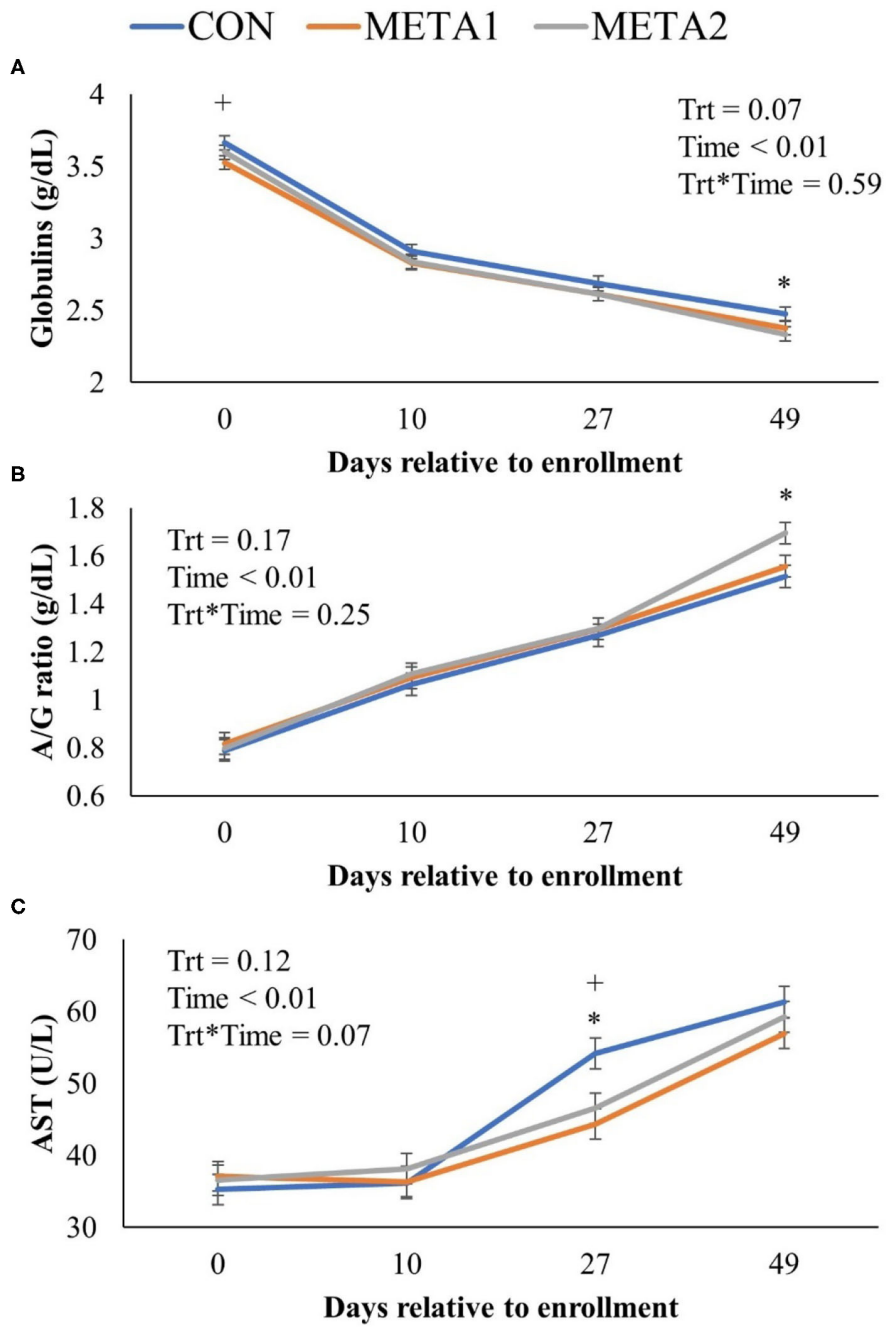
Effect of metaphylaxis on blood concentration of globulins **(A)**, albumin to globulin ratio **(B)**, and aspartate aminotransferase **(C)**. A cross (+) or asterisk (^*^) indicates a *P* < 0.05 when comparing CON with META1 or META2, respectively. Calves enrolled in META1 received single SQ injection of tildipirosin (4 mg/kg) at enrollment, calves enrolled in META2 received one SQ injection of tildipirosin at enrollment and a subsequent SQ tildipirosin injection 17 days after the first injection, and CON calves remained untreated.

The effect of tildipirosin metaphylaxis on blood chemical panel variables is presented in Table 3. Metaphylaxis did not influence the concentration of the blood analytes assessed. However, CON calves tended to have increased circulating concentration of globulins throughout the study period compared to META1 and META 2 calves (*P* = 0.07, Table 3). Additionally, the dynamics of the serum concentration of globulin, albumin to globulin ratio, and aspartate aminotransferase by day of sampling are illustrated in Figure 3. Calves enrolled in the CON group calves had increased serum concentration of globulins in comparison to META1 calves at enrollment, and META2 calves at 49 days after enrollment (Figure 3A; *P* < 0.01 and *P* = 0.01, respectively). Additionally, serum albumin to globulin ratio was only increased for META2 calves in comparison to CON counterparts at 49 days after enrollment (Figure 3B; *P* < 0.01). Aspartate aminotransferase serum concentration was greater for CON calves than for META1 and META2 calves at 27 days after enrollment (Figure 3C; *P* < 0.01 and *P* = 0.01, respectively).

**Table 3:** Effect of tildipirosin metaphylaxis on ruminant blood chemical panel variables.

**Variable**	**Treatment**			***P***		
	**CON^1^**	**META1^2^**	**META2^3^**	**TRT**	**Time**	TRT^*^Time
Total protein (g/dL)	6.19	6.10	6.11	0.12	<0.01	0.70
Albumin (g/dL)	3.27	3.27	3.27	1.00	<0.01	0.85
Calcium (mg/dL)	10.7	10.7	10.6	0.29	<0.01	0.66
Phosphorus (mg/dL)	9.31	9.36	9.27	0.50	<0.01	0.37
Glucose (mg/ dL)	114.9	113.1	113.1	0.46	<0.01	0.27
BUN^4^ (mg/dL)	11.5	11.3	11.4	0.74	<0.01	0.83
Creatinine (mg/dL)	0.81	0.82	0.81	0.93	<0.01	0.67
Bilirubin (mg/dL)	0.21	0.22	0.23	0.21	<0.01	0.80
CK^5^ (U/L)	121	114	122	0.48	<0.01	0.32
AST^6^ (U/L)	46.7	43.7	45.1	0.12	<0.01	0.07
Globulins (g/dL)	2.93	2.84	2.85	0.07	<0.01	0.59
A/Gy^7^	1.16	1.19	1.22	0.17	<0.01	0.26
GGT^8^ (U/L)	157.7	148.4	154.6	0.74	<0.01	0.41
GLDH^9^ (U/L)	39.8	34.5	38.7	0.37	<0.01	0.21
Magnesium (mEq/L)	1.93	1.94	1.93	0.36	<0.01	0.45
Sodium (mEq/L)	139.3	139.4	139.3	0.96	<0.01	0.49
Potassium (mEq/L)	5.57	5.53	5.53	0.46	<0.01	0.36
Chloride (mEq/L)	100.5	100.4	100.6	0.78	<0.01	0.48
Na/K^10^ (mEq/L)	25.1	25.3	25.3	0.40	<0.01	0.11

1 *CON: untreated controls*.

2 *META1: single SQ injection of tildipirosin (4 mg/kg) at enrollment*.

3 *META2: one SQ injection of tildipirosin at enrollment and a subsequent SQ tildipirosin injection 17 days after the first injection*.

4 *BUN: blood urea nitrogen*.

5 *CK: creatine kinase*.

6 *AST: aspartate aminotransferase*.

7 *A/G: albumin to globulin ratio*.

8 *GGT: gamma-glutamyl transferase*.

9 *GLDH: Glutamate dehydrogenase*.

10 *Na/K: sodium to potassium ratio*.

## Discussion

Transportation of dairy calves to an off-site calf raising facility has become a common management strategy for many dairy enterprises (26, 27). Approximately 10% of heifer calves born in the United States are transported for long distances to be raised in specialized facilities and commingled with other calves from different sources (22). Among these calves, the risk for BRD is elevated, and the use of metaphylaxis is often utilized to control BRD. The long-acting macrolide tildipirosin has desirable properties for BRD metaphylaxis in high risk cattle because it has a long half-life leading to a sustained concentration of the macrolide in lung tissue and bronchial fluid (23). Previous studies have evaluated the efficacy of metaphylactic use of tildipirosin to prevent BRD in group housed pre-weaned dairy calves (9), and in calves transported to a veal facility (13). Furthermore, other studies evaluated the development of lung lesions and other measures of health in animals submitted to microbial challenges after metaphylactic administration of tildipirosin (28, 29). To the best of our knowledge, metaphylactic approaches to high-risk dairy calves housed in individual hutches has not been fully investigated.

Metaphylaxis did not decrease the incidence of BRD during the pre-weaning period. We only observed a numerical decrease in BRD incidence for calves that received metaphylaxis. In veal calves, metaphylactic treatment using tildipirosin 12 days after arrival was not associated with the number of BRD treatments (13). However, others have reported that metaphylaxis can improve respiratory tract health of pre-weaned calves. Teixeira et al. (10) showed that tildipirosin metaphylactic injections decreased the likelihood of BRD in pre-weaned calves housed in group pens. Moreover, metaphylactic injection of tildipirosin 5 days prior to *Histophilus somni* inoculation decreased the presence of this bacterium in bronchial secretion samples collected three days after challenge (29). The BRD incidence in our study calves was lower than we expected. For instance, two recent studies showed BRD incidences of ~22% for calves being diagnosed and treated at least once in the first 3 months of age (4, 19). It is plausible that the lack of impact of metaphylaxis on BRD incidence might be due to the low BRD incidence and consequently low statistical power of our study. Perhaps transportation in our study was not as stressful as initially assumed, and the calves were not in high risk of BRD as we had expected. Because calves were housed in individual hutches after arrival, there was not close contact between them, and it is likely that pathogen transmission between calves was reduced during the pre-weaning period, which resulted in low BRD incidence. Our metaphylactic strategies were designed based on the BRD incidence curve that was built during study design (Figure S1), and injection were administered close to the peaks of BRD incidence during the pre-weaning period. In feedlots, the use of epidemiologic curve plots is helpful to determine a temporal pattern of diseases and may influence management strategies such as metaphylaxis (30). Berman et al. (13) also determined their metaphylactic injection timing (3 weeks after veal calves' arrival) based on the expected BRD incidence peak. Because they also reported a lower than expected BRD incidence, they highlighted the importance of a cohort risk assessment before the development of metaphylactic treatment protocols (13).

Like BRD incidence, lung health assessed by thoracic ultrasonography at weaning was not influenced by metaphylaxis in our study. Thoracic ultrasonography is an accurate and practical diagnostic tool for BRD-related lung lesions in calves (31), and it could represent BRD cases that did not manifest in clinical signs evaluated by the researchers. Berman et al. (13) also did not observe a reduction in lung lesions diagnosed by thoracic ultrasonography in veal calves that received metaphylactic injection of tildipirosin. However, studies involving pathogen-challenges reported that metaphylaxis with tildipirosin improved lung health when assessed through thoracic ultrasonography. Heifers that received a tildipirosin injection 10 days prior to *Mannheimia haemolytica* challenge had decreased lung lesion scores than heifers that received tulathromycin injection or negative saline controls (28). Furthermore, lung lesions were less severe for calves that received tildipirosin injection 5 days prior to *Histophilus somni* inoculation, with a lack of necrosis and only areas of acute bronchopneumonia surrounded by normal lung tissue (29).

Additionally, we observed that mortality during the pre-weaning period tended to be reduced in calves enrolled in the META2 treatment group compared to CON calves. In general, the pre-weaning mortality average was 1.2%, which is considerably lower than the mortality rates previously described. For instance, the overall mortality of calves during the pre-weaning period in the United States has been recently reported to be 5.0%, according to the latest USDA National Animal Health Monitoring Survey (3). Others have reported pre-weaning mortality in herds located in New Mexico, California, and Minnesota to be 14% (range from 7.0 to 29.1%), 2.8% (range from 1.7 to 7.2%) and 3.5% (range from 0 to 10%), respectively (4, 19, 32). In contrast to our results, the metaphylactic use of tildipirosin did not impact mortality in group-housed pre-weaned calves (9).

Growth during the pre-weaning period of dairy calves is affected by BRD (9, 27). Hence, strategies to mitigate BRD during the pre-weaning period can potentially result in improved weight gain of calves. Additionally, calves that received tildipirosin metaphylaxis prior to a *Mannheimia haemolytica* respiratory challenge had greater feed consumption during the 3-day observation period after inoculation, suggesting that metaphylaxis could potentially increase growth; however the animals were euthanized for data collection purposes and no conclusions in long-term ADG could be made (28). Because BRD incidence was not reduced by metaphylaxis in our study, it is not surprising that growth was also not influenced. Others have also reported that metaphylactic administration of tildipirosin had no effect on ADG of calves during the pre-weaning period (9, 13).

Some biomarkers of inflammation that have been previously associated with BRD or stress were affected by metaphylaxis. For instance, Hp and SAA are acute phase proteins that are elevated in blood in calves that show clinical signs of BRD (33–35). Even though metaphylaxis did not decrease BRD incidence in our study, the concentration of Hp and serum-amyloid A was decreased in META2 calves in comparison with CON calves at 27 days after enrollment. Furthermore, animals from META2 group had decreased concentrations of AST and decreased neutrophil to lymphocyte ratio in comparison to CON calves. Additionally, CON group animals had increased concentration of globulins and lower albumin to globulin ratio at weaning in comparison to META1 and META2 calves. Neutrophil to lymphocyte ratio has been used as a measurement of ruminant stress (36). Calves diagnosed with BRD are reported to have increased levels of AST (37), increased serum globulin concentrations (38) and decreased albumin in comparison to healthy calves (35). Collectively, blood analysis results suggest that even though the clinical disease was not influenced by metaphylaxis, systemic inflammatory state of calves were improved.

In conclusion, metaphylactic use of tildipirosin did not decrease BRD incidence, prevalence of lung lesions diagnosed by ultrasonography at weaning, nor it had an impact on growth during the pre-weaning period of dairy calves transported to a heifer raising facility. However, mortality tended to be lower in calves enrolled in the META2 treatment groups, and systemic inflammation status of calves were improved by metaphylaxis based on circulating biomarkers of inflammation and stress. Given the concern regarding antimicrobial resistance development and judicious use of antimicrobial drugs, our results do not support the metaphylactic use of tildipirosin in field conditions with already low incidence of BRD morbidity and mortality as described herein. However, even with low incidence of disease, metaphylaxis tended to decrease mortality by 60% (1.5 vs. 0.6), and improved the inflammatory status of calves, one could speculate that it could be an efficacious strategy to control BRD and improve welfare in herds where BRD incidence is greater than reported herein. Hence, we believe that more research is needed to evaluate potential benefits of metaphylaxis in herds where the incidence of BRD and mortality are greater than what was observed in our study.

## Data Availability Statement

The raw data supporting the conclusions of this article will be made available by the authors, without undue reservation.

## Ethics Statement

The animal study was reviewed and approved by all activities performed in this study were reviewed and approved by the Texas Tech University Institutional Animal Care and Use Committee (#18081-10). Written informed consent was obtained from the owners for the participation of their animals in this study.

## Author Contributions

The study was designed by VM, TB, RN, and MB. Data collection was conducted by MC, LF, PM, DP, TR, and TS. Database compilation was done by MC, and data analysis was done by VM. The manuscript was drafted by MC and VM, which was then reviewed by all authors. The research protocol was developed with input of all authors. All authors contributed to the article and approved the submitted version.

## Conflict of Interest

This research was funded by a grant from Merck Animal Health Inc., which employed TB. This co-author participated in the study design and reviewed the manuscript, but did not participate in the collection, analysis and interpretation of data. The remaining authors declare that the research was conducted in the absence of any commercial or financial relationships that could be construed as a potential conflict of interest.
