# Supplementary material for: The Effect of Metaphylactic Use of Tildipirosin for the Control of Respiratory Disease in Long-Distance Transported Dairy Calves
Source: Front Vet Sci. 2020 Sep 8;7:632. doi: 10.3389/fvets.2020.00632 (PMC7506132; doi:10.3389/fvets.2020.00632)

Figure S1: The distribution of BRD cases that occurred from April of 2017 to April of 2018 for pre-weaned dairy heifers that were transported within the first week of life from dairies in Minnesota to a calf ranch in New Mexico.

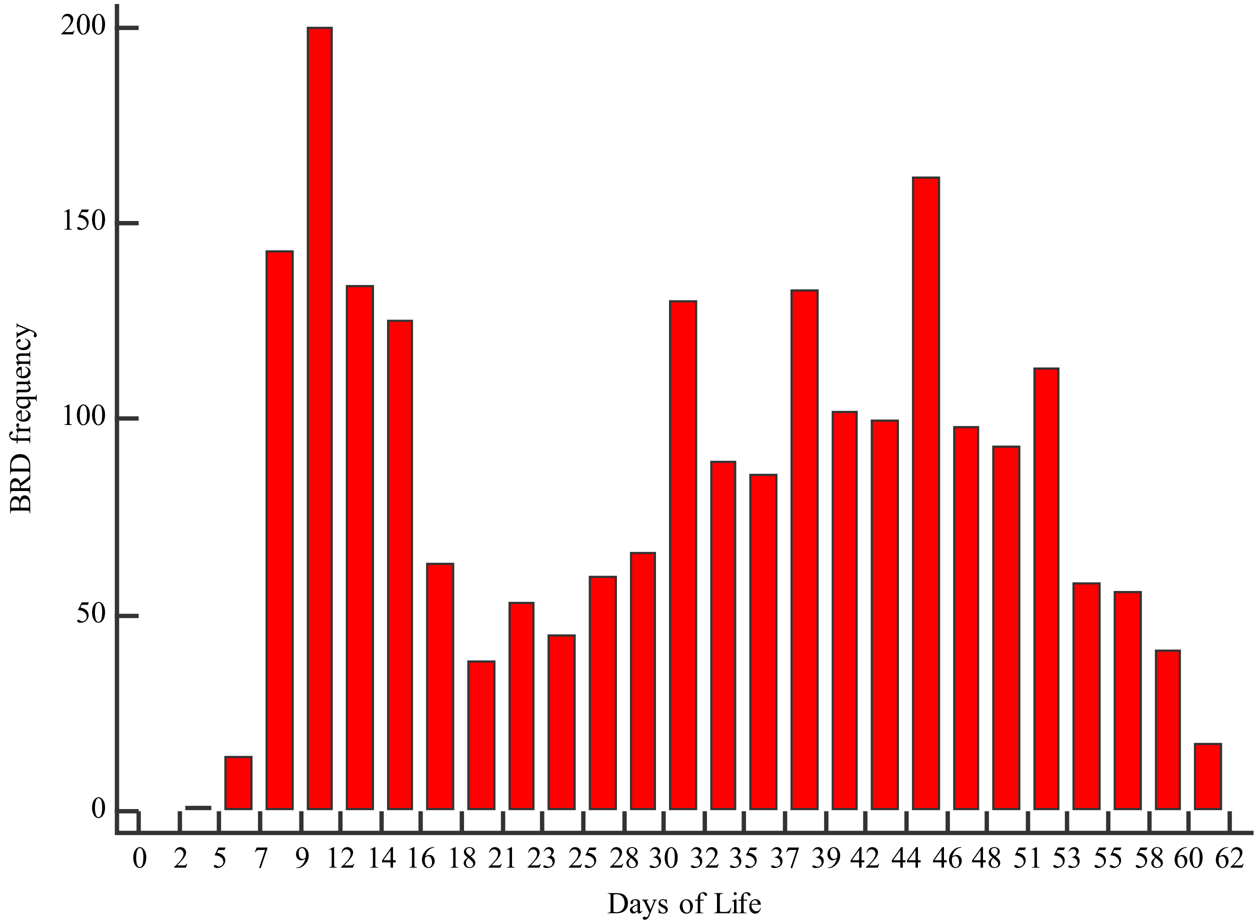

Supplement: Supplementary file 1 [file Image_1.pdf]
